# Mast cells in human choroid and their role in age-related macular degeneration (AMD)

**DOI:** 10.1007/s10238-024-01361-9

**Published:** 2024-05-10

**Authors:** Domenico Ribatti, Rosanna Dammacco

**Affiliations:** Department of Translational Biomedicine and Neuroscience, University of Bari Medical School, Bari, Italy

**Keywords:** Age-related macular degeneration, Angiogenesis, Inflammation, Mast cells

## Abstract

The role of mast cells in physiologic and pathological processes extends far beyond the allergy processes: they are involved in wound healing, chronic inflammation, and tumor growth. This short article emphasizes the role played by mast cells in age-related macular degeneration (AMD). Mast cells can induce angiogenesis and are present around Bruch's membrane during the early and late stages of choroidal neovascularization in AMD. Proteolytic enzymes released by mast cells lead to thinning of the choroid in AMD as well as degradation of vascular basement membranes and Bruch's membrane, which in turn could result in retinal pigment epithelial death and choriocapillaris degeneration in geographical atrophy and exudative AMD.

## Introduction

Mast cells, first identified and described by Paul Ehrlich in his doctoral thesis in 1878 [3], originate from progenitor cells in the bone marrow, move through the circulation, and become mature after homing to different organs under the influence of the local microenvironment [7, 21]. Mast cells preferentially reside near surfaces exposed to environmental triggers, such as skin, airways, and gastrointestinal and urogenital tracts [22].

Mast cells have multiple roles extending beyond their classical role in IgE-mediated allergic reactions. When mast cells are activated, they degranulate releasing a wide range of already stored mediators, including histamine, serotonin, tumor necrosis factor alpha (TNF-α), proteoglycans and various proteases, and/or secrete newly synthesized lipid derivatives, cytokines, and chemokines. In this context, mast cells are involved in the regulation of the functions of many organs and tissues.

Although these cells secrete many pro-inflammatory agents, they also release many anti-inflammatory agents. In this context, mast cells can change from protective immune cells to potent pro-inflammatory cells which influence the progression of many pathological conditions, including inflammatory diseases, autoimmune diseases, and tumors.

### Inflammation in age-related macular degeneration

Age-related macular degeneration (AMD) is a multifactorial disease, including genetic predisposition, oxidative stress, neovascularization, inflammatory responses, and remodeling processes of the retinal extracellular matrix, characterized by progressive degeneration of the photoreceptor/retinal pigment epithelium/choriocapillaris complex primarily in the macular region of the eye, resulting in irreversible central vision loss.

There are mainly two types of AMD: dry (also named non-neovascular, non-exudative, or atrophic) AMD and wet (also named neovascular or exudative) AMD (nAMD) [17]. Dry AMD is characterized by the increase of extracellular deposits called drusen, along with advanced-stage geographical atrophy which is characterized by decreasing retinal pigment epithelium cells, photoreceptors, and choroidal capillaries. Wet AMD is characterized by choroidal neovascularization, leading to severe and fast vision impairment, accompanied by hard exudate, leaking fluid or retinal hemorrhage, retinal pigment epithelium detachment, or fibrosis around neovascular tufts [17].

Literature evidence suggests that inflammation plays an important role in its pathogenesis. Leukocytes are closely correlated with AMD pathogenesis, and both innate and adaptive immune cells play key roles in AMD [2]. Innate immune cells (macrophages, dendritic cells, and neutrophils) can stimulate adaptive immune cells (B cells and T cells) and participate in choroid neovascularization pathogenesis []. The levels of pro-inflammatory factors such as interleukin (IL)-6, IL-8, monocyte chemotactic protein-1 (MCP-1), and vascular endothelial growth factor (VEGF) are elevated in the intraocular fluid of patients with dry and wet AMD. Cytokines, including VEGF, IL-1β, IL-6, IL-8, IL-10, IL-17, transforming growth factor beta (TGF-β), and TNF-α, have angiogenic properties, whereas IL-4, IL-12, and interferon beta (IFN-β) inhibit angiogenesis.

### Mast cells in the eye

In the eye, mast cells are present in the uveal tract, iris, ciliary body, and choroid of different animal species [13]. In humans, mast cells are abundant in the anterior and posterior uvea but are absent in the retina [8, 11]. Within the choroid, mast cells have a characteristic periarteriolar distribution [13], primarily along the long posterior ciliary arteries (LPCA) and their branches [13].

In the 1960s, Enerbäck described two morphologically distinct subpopulations of rodent mast cells, based on their specific staining characteristics and preferential tissue homing, i.e., connective tissue mast cells (CTMCs) present in the connective tissues, and mucosal mast cells (MMCs) located on the mucosae of the respiratory and gastrointestinal tracts. CTMCs could be distinguished from MMCs by red staining with safranin due to the presence of large amounts of heparin in their secretory granules. After appropriate fixation and sequential staining with Alcian blue and safranin, MMCs stain blue, being thus differentiated from CTMCs which stain with safranin and are red. Differential affinity for Alcian blue can be visualized with sequential staining, consisting of Alcian blue followed by safranin [20]. Histochemical studies have revealed rat choroidal mast cells are of the connective tissue phenotype, containing mixed Alcian blue/safranin-positive granules. Transmission electron microscopy confirms these findings by revealing rat choroidal mast cell granules to be of uniform diameter [13].

In humans, mast cells may be classified on the presence in their granules of high levels of tryptase but little or no chymase (MCT) in intestinal and pulmonary mucosa predominantly found at mucosal sites, or mast cells containing chymase, and little or no tryptase (MCC), and finally, mast cells containing tryptase, chymase, and carboxypeptidase (MCTC) predominantly found in the skin, lymph nodes, and lung and gut submucosa [6]. May [11] showed that most of the mast cells in the normal human choroid are of the MCTC variety, whereas McLeod et al. [15] found that MCT was the predominant variety in the choroid in aged eyes with and without AMD. May [11] identified chymase- and tryptase-positive mast cells in the human uvea and studied their association with different types of resident uveal cells. Most of the choroidal mast cells contain both chymase and tryptase, in agreement with in vitro studies of choroidal cell suspensions [16]. In contrast, mast cells of ciliary and sphincter pupillae muscle express a specific, tryptase-positive, chymase-negative protease profile [11]. The human iris contains only a few mast cells. In rodents, the number of iris mast cells is even lower than in primates and humans [13]. On the other hand, in the human choroid, many mast cells are in the inner part toward the capillary layer. This difference in mast cell distribution coincides with characteristic structures of the vascular walls in both tissues. The vessels of the iris have a thick, perivascular sheath forming a barrier for larger molecules such as proteins. In contrast, the choriocapillaris is highly fenestrated, and antigenic substances can easily pass into the choroidal stroma. In this respect, the high number of mast cells in the inner choroid adjacent to the choriocapillaris might be necessary for an appropriate antigenic reaction.

### Mast cells in AMD

Increased numbers of mast cells have previously been reported in the choroid of individuals with AMD [1]. Mast cells can induce angiogenesis and are present around Bruch's membrane during the early and late stages of choroidal neovascularization in AMD [18]. Proteolytic enzymes released by mast cells lead to thinning of the choroid in AMD as well as degradation of vascular basement membranes and Bruch's membrane, which in turn could result in retinal pigment epithelial death and choriocapillaris degeneration in geographical atrophy and exudative AMD [14]. Finally, mast cell releases angiogenic factors and proteases that promote vascularization, including VEGFA, fibroblast growth factor-2 (FGF-2), IL-8, nerve growth factor (NGF), TGFβ, TNFα, heparin, histamine, tryptase, chymase, MMP-2, and MMP-9 [19] that may contribute to neovascularization occurring in AMD.

### Therapeutic approach

Intravitreal injections of anti-VEGF agents, such as ranibizumab [4] and aflibercept [5], have been widely and effectively used worldwide in the clinical treatment of nAMD via targeting choroidal neovascularization. Mast cells secrete VEGF, a potent inducer of neoformation of blood vessels and named vascular permeability factor (VPF), responsible for increased vascular permeability. Nevertheless, about one-third of patients do not get effects from anti-VEGF therapy owing to macular fibrosis or atrophy [9].

The fact that inflammation appears early in AMD pathology may explain why anti-inflammatory agents are beneficial as preventive or adjunctive therapies in combination with anti-VEGF therapy. Mast cells stimulate inflammation through the release of TNF-α, which in turn, recruits inflammatory cells including macrophages and neutrophils. Moreover, mast cells promote dendritic cell migration to lymph nodes through TNF-α, histamine, and IL-6 and promote anti-tumor T-cell phenotypes through histamine. In this context, although anti-VEGF factor therapy is the first line of defense against neovascular AMD, anti-inflammatory agents such as corticosteroids, nonsteroidal anti-inflammatory drugs (NSAIDs), immunosuppressive agents (e.g., methotrexate and rapamycin), and biologics (e.g., infliximab, daclizumab, and complement inhibitors) may provide an adjunct or alternative mechanism to suppress the inflammatory processes in AMD.

More specifically, stabilization of mast cell degranulation is another therapeutic strategy for targeting mast cells and preventing or abrogating their activation. Disodium cromoglycate, which inhibits mast cell degranulation, lowered the rate of serous retinal detachment from 80 to 16% when it was administered before the degranulation-activating compound. Tranilast, an anti-allergic drug that inhibits the release of chemical mediators from mast cells, suppresses laser-induced choroidal neovascularization in the rat [23].

## Concluding remarks

Recently, it has been established that mast cells play a crucial role in AMD. Choroidal mast cells release different mediators, including histamine, cytokines, and proteases. Proteases, including tryptase, MMPs, and granzyme B, disrupt extracellular matrix leading to the degradation of the choriocapillaris basement membrane, endothelial damage, and vascular dropout [12]. Moreover, choroidal mast cells are located near the choroidal vasculature, intervening in the modulation of angio-inflammatory processes [10]. Finally, mast cells through the secretion of TNF-α recruit neutrophils and macrophages, amplifying the inflammatory response. The number of mast cells may be reduced by the targeted induction of apoptosis or by blocking their recruitment, migration, and differentiation. Many pharmacological agents have been developed that modulate mast cell functions. They block mediator receptors on target cells, including H1 receptor antagonists, CysLT1 receptor antagonists, and PGD_2_ receptor antagonists; inhibit mast cell mediator synthesis, including omalizumab, disodium cromoglycate, and imatinib; block mast cell activation or mediator release, including steroids and NSAIDs. In this context, the inhibition of  mast cell activation and of the release of inflammatory mediators may be considered a novel therapeutic strategy in treating AMD.
